# Histone acetyl transferase TIP60 inhibits the replication of influenza a virus by activation the TBK1-IRF3 pathway

**DOI:** 10.1186/s12985-018-1079-3

**Published:** 2018-11-08

**Authors:** Guoyao Ma, Lin Chen, Jing Luo, Bo Wang, Chengmin Wang, Meng Li, Chengmei Huang, Juan Du, Jiajun Ma, Yungfu Chang, Hongxuan He

**Affiliations:** 1 National Research Center for Wildlife Borne Diseases, Institute of Zoology, Chinese Academy of Sciences, No.1-5 Beichenxilu, Chaoyang District, Beijing, 100101 People’s Republic of China; 2 University of the Chinese Academy of Sciences, Beijing, 100101 China; 3 Cornell University College of Veterinary Medicine, Ithaca, NY 14853 USA

**Keywords:** Histone acetyl transferase TIP60, Influenza a virus, Nucleoprotein, Polymerase activity, Type I interferon

## Abstract

Influenza A virus (IAV) is an important pathogen that poses a severe threat to the health of humans. Nucleoprotein (NP) of IAV plays crucial roles in the viral life cycle by interacting with various cellular factors. Histone Acetyl Transferase TIP60 is a key target of several viral proteins during infection, including HIV-1 Tat, HPV E6, HTLV-1 p30^II^ and HCMV UL27 proteins. However, Whether the interaction between the IAV NP and TIP60, and the role of TIP60 in IAV life cycle are largely unknown. Here, we showed that IAV infection up-regulated TIP60 protein and RNA expression. Overexpression of TIP60 inhibited viral protein and RNA expression and reduced the progeny viral titer. Further study revealed that TIP60 inhibited viral replication through activation of TBK1-IRF3 signaling pathway. Furthermore, we demonstrated that the NP protein of IAV interacted with TIP60. Together, these results indicate that TIP60 play a repressor in IAV infection, and it may be a possible target for antiviral drugs.

## Background

Influenza A virus (IAV) is a critical respiratory pathogen that belongs to the Orthomyxoviridae family**,** which causes severe morbidity and mortality rates every year [1]. IAVs have negative-stranded RNA genomes consisting of eight RNA segments, and encode up to 17 proteins: hemagglutinin (HA), neuraminidase (NA), nucleoprotein (NP), polymerase basic protein 1(PB1), polymerase basic protein 2 (PB2), PB1-F2, PB1-N40, polymerase acidic protein (PA), PA-X, PA-N155, PA-N182, matrix protein 1 (M1), M2 and M42, nonstructural protein1 (NS1), NS2, NS3 [2–8]. NP is a viral RNA genome-encapsulating structural protein which can associates with viral RNA and three polymerase proteins (PB1, PB2 and PA) to form viral ribonucleoprotein (vRNP) complex [9]. NP also interacts with a plethora of cellular factors in viral RNA replication, virus assembly and intracellular trafficking of the virus genome [10, 11]. Our previous study demonstrated NP interacted with histone deacetylase 1, which downregulated the acetylation level of NP. While the interaction between the NP and Histone Acetyltransferase (HATs) enzymes were largely unknown.

HATs are an enzyme family that regulates these processes by catalyzing the transfer of an acetyl moiety onto target proteins. TIP60 is a member of the MYST (MOZ, Ybf2/Sas3, Sas2, and TIP60) family of histone acetyltransferase that was found by interaction with the HIV-1 Tat protein [12, 13]. It contains an N-terminal chromodomain and a C-terminal conserved MYST domain (HAT domain and Cys-Cys-His-Cys zinc finger). HAT domain binds to acetyl coenzyme A and the substrate, and the zinc finger is required for protein–protein interactions [14]. Expression of TIP60 is observed in multiple cell lines and tissues, and its homologues have been identified in chicken, mouse, and human [15].

TIP60 acts as a coactivator or a co-repressor involved in transcriptional regulation of a variety of factors, such as myelocytomatosis oncogene c (c-Myc), signal transducers and activators of transcription 3 (STAT3)[16, 17]. In DNA damage response, TIP60 was activated and it interacts with the kinase ataxia telangiectasia-mutated (ATM), which activates and induces ATM autophosphory resulting in initiates a series of reparative reactions [18]. TIP60 is a component of the p53 pathway. It was required for both cell growth arrest and apoptosis mediated by p53 and also induces its acetylation [19]. It can regulate the stability of p53 by interfering with Mdm2-mediated p53 degradation to maintain a basal pool of p53 in normal growth conditions, but TIP60 functions as p53 co-activator following DNA damage [20]. Moreover, a variety of cellular factors involved in the apoptosis mediated by p53 as cofactor. Programmed cell death 5 (PDCD5) binds to TIP60 and enhances HAT activity of TIP60 and induces TIP60-dependent K120 acetylation of p53, which advances expression of apoptosis-related genes [21]. ING5 can as a cofactor of TIP60 to involved in the acetylation of p53 at K120 and it formed a complex with p53 and TIP60 [22]. USP7 interacts with and deubiquitinates TIP60 to enhance its stability, which contribute to activated of the P53-mediated apoptotic pathway [23]. In addition, TIP60 functions as a tumor suppressor was widely accepted and it lower expression was observed in multiple cancers [24].

In addition to HIV type 1 (HIV-1) Tat, TIP60 also is a key target of several viral proteins during infection. Human T cell lymphotropic virus type 1 (HTLV-1) p30^II^ enhances Myc-associated transcriptional and transforming activities through interaction with the HAT of TIP60 [25]. Furthermore**,** TIP60 can interact with viral E6 and UL27 proteins, which encoded by human papillomavirus (HPV) and human cytomegalovirus (HCMV), respectively [26, 27]. However, whether the TIP60 interacted with NP of IAVs is largely unknown.

In this study, we showed that the NP protein of IAV interacted with TIP60. We found that IAV infection upregulated the protein expression of TIP60 in a time- and dose-dependent manner. We further examined the effects of TIP60 on IAV replication. Overexpression of TIP60 inhibited viral replication through activation of TBK1-IRF3 signaling pathway.

## Methods

### Cells, viruses, and plasmids

A549 cells, 293 T cells and MDCK cells were grown and maintained in Dulbecco’s modified Eagle’s medium (DMEM) supplemented with 10% fetal bovine serum (FBS) and 1% penicillin-streptomycin in a 5% CO_2_ incubator of 37 °C. A/Qing Hai/environment/2005 (H5N1) strain were propagated in 10-day-old specific pathogen-free (SPF) embryonic chicken eggs and titrated on MDCK cells. All experiments with IAVs were carried out in biosafety level 3 containment laboratories approved by the Chinese Academy of Science.

### Plasmids construction

The full-length TIP60 was cloned into the pCDNA3.0-HA vector. NP gene from A/Qing Hai/environment/2005(H5N1) influenza virus was cloned into pcDNA3.1-Myc and pHW2000 vectors as previously described [28]. The sequence integrity of all plasmids was identified by gene sequenced.

### Transfection and virus infection

Cells (4 × 10^5^) were spread in 6-well plates and incubated for 18 h. Cells were transfected at 80–90% confluence with Lipofectamine 3000 reagent (Life Technologies) according to the manufacturer’s guidelines. After 48 h of incubation, the Cells were infected with the indicated virus at a multiplicity of infection(MOI) of 1. The DMEM supplemented for virus infection contained 2% FBS and 2.5% bovine serum albumin (BSA). Incubation 1 h at 37 °C and shaken at every 15 min, removed the inoculum and washed once with PBS. Then, added to fresh serum-free DMEM containing 2.5% BSA and incubated back at 37 °C.

### TCID_50_

One day before infection, 3 × 10^4^ MDCK cells were seeded in a 96-well plate with five repetitions. 24 h later, cells were infected with different dilutions of virus for 1 h at 37 °C, with shaking every 15 min. The inoculum were removed and washed once with PBS. Then, added to fresh serum-free DMEM containing 2.5% BSA and incubated back at 37 °C.The cytopathy was observed for one time every 12 h and confirmed by the hemagglutination assay. Calculated the TCID_50_ by the Reed-Muench method.

### RNA extraction and quantification real-time PCR

Total RNA was extracted from infected cells using TRIzol reagent according to the manufacturer (Invitrogen), and 2 μg of RNA was reverse-transcribed into cDNA using the GoScript Reverse Transcription System (Promega). Primers used for real-time RT-PCR are TIP60 primers forward CAGGACAGCTCTGATGGAATAC and reverse AGAGGACAGGCAATGTGGTGAG. The primers of the corresponding virus mRNA, cRNA and vRNA are described in previous studies [1]. Fold change in RNA levels were calculated using the delta**–**delta threshold cycle (***ΔΔ***CT) method as described elsewhere [29].

### Western blotting

First, cells were washed PBS and harvested in lysis buffer (pH 7.6, 50 mM Tris-HCl, 150 mM NaCl, 0.1% SDS, 1% Triton-100, and protease inhibitor [Roche]). Next, the total cell lysates were quantitated by using a BCA kit (APPLYGEN)**.** Then, the equal amounts of proteins obtained were subjected to SDS- PAGE, and resolved proteins were transferred onto nitrocellulose membranes. Subsequently, after blocking with 5% nonfat milk, the membranes were incubated with primary antibody followed by secondary antibody. The membrane blots were visualized by using the Dura chemiluminescent kit (Millipore), and images were acquired on ImageQuant las 4000 imaging system (GE Healthcare).

### Luciferase reporter assay

All polymerase complex component plasmids (PB2/pHW2000, PB1/ pHW2000, PA/ pHW2000, and NP/pHW2000) DNA (1 μg each) and a luciferase RNA expression (vNS-luc/pHH21) vector and pRL-TK (50 ng) were cotransfected into 293 T cells. At the same time, pcDNA3.0-HA empty vector or plasmid expressing TIP60 was also transfected into 293 T cells. The Renilla luciferase was used as an internal control. At 36 h post-transfection, cells were lysed and measured luciferase activity using the Dual Luciferase Assay System (Promega).

### Co-immunoprecipitation analysis

293T cells were transfected with pCDNA3.1-MYC-NP and pCDNA3.0-HA-TIP60 or pCDNA3.0-HA. At 48 h post-transfection, cells were lysed in binding buffer containing protease inhibitor cocktail. The cell lysates were incubated with anti-MYC at 4 °C for 6–8 h. Then, added to protein G-Sepharose beads incubated at 4 °C overnight. Beads were washed three times with binding buffer, boiled with 2× SDS loading buffer for 10 min. Subsequently, the samples were assayed with MYC and HA antibody using western blot.

### Immunofluorescence analysis

293T or A549 cells were grown on glass coverslip and transiently transfected with plasmids expressing TIP60. 48 h after transfection, the cells were infected with virus at a MOI of 3. At 12 h p.i., cells were fixed with 4% paraformaldehyde for 20 min, washed three times with PBS, and permeabilized with 0.1% Triton X-100 in PBS for 10 min. The cells were then washed with PBS three times and blocked with 1% bovine serum albumin in PBS at RT for 10 min. Cells were incubated overnight at 4 °C with primary antibodies, then washed three times with PBS and incubated with the secondary fluorochrome-conjugated antibodies for 1 h. After several washes, cells were counterstained with DAPI (Sigma) for 3 min, and analyzed by confocal laser microscopy (Leica 780).

### Statistic analysis

All date were analyzed with software GraphPad Prism 6 (GraphPad Prism, La Jolla California USA). The date were expressed as mean **±** SD. Statistical comparisons were made by Student’s-test. AP value < 0.05 was considered significant.

## Results

### Influenza infection increases TIP60 mRNA and protein expression

**Fig. 1:**
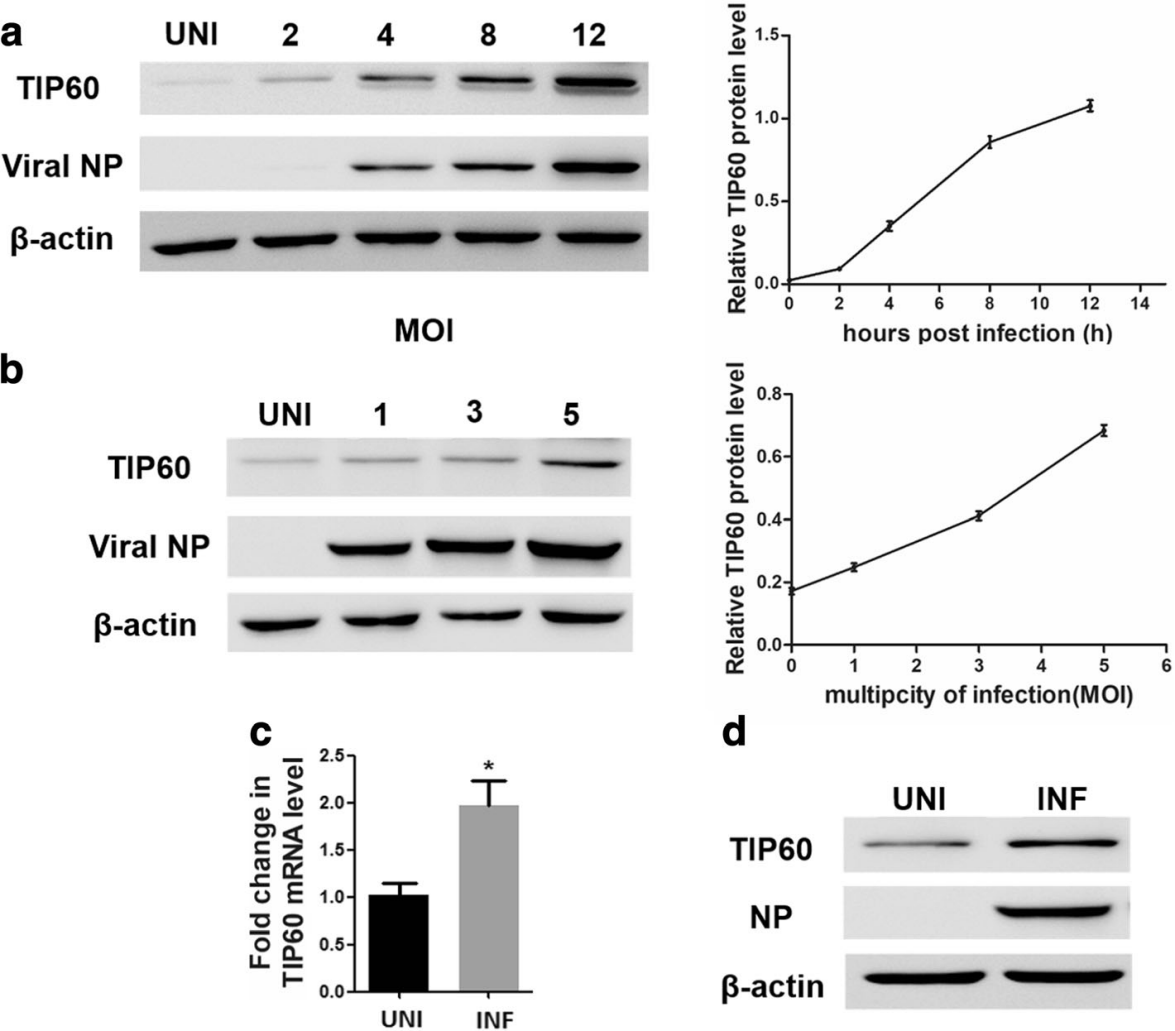
IAV upregulated the mRNA and protein expression of TIP60. **a** Lung epithelial A549 cells were infected with IAV virus (MOI **=** 1). Cells were harvested 2, 4, 8, and 12 h post-infection and analyzed by western blotting with anti-TIP60 and anti- NP antibodies. **b** A549 cells were infected with IAV virus at indicated MOIs and harvested at 12-h post infection for Western blot analysis of TIP60, NP and Actin. The TIP60 and Actin protein bands were quantified using Image J software, and the amount of TIP60 was normalized to Actin. **c** A549 cells (4 × 10^5^) were mock-infected or infected with IAV at an MOI of 1. The uninfected (UNI) and infected (INF) cells were harvested 12 h post-infection and analyzed by qRT-PCR. Data are mean **±** SD of three independent experiments. Two-way ANOVA test; *p < 0.05. **d** 293 T cells were infected with IAV virus (MOI **=** 1). At 12 h p.i, cells were harvested and analyzed by western blotting

To explore the expression of TIP60 in response to IAV infection, A549 cells were infected with IAV at an MOI of 1. The protein expression of TIP60 was detected by western blotting at different time points post infection. The results showed a gradual increase in TIP60 protein expression with progression of infection (Fig. 1a). In addition, TIP60 protein level was elevated by IAV in a dose-dependent manner (Fig. 1b).

Subsequently, the mRNA level of TIP60 was further evaluated after IAV infection. A549 cell cultures were collected to measure TIP60 mRNA expression by qRT-PCR at 12 h post-infection. The results revealed a remarkable 2-fold increase in TIP60 mRNA levels compared with the mock (Fig. 1c). IAV infection also resulted in increased TIP60 protein levels in 293 T cells (Fig. 1d). Taken together, these data demonstrated that IAV infection upregulated TIP60 expression.

### Host factor TIP60 reduced the progeny viral titer

**Fig. 2:**
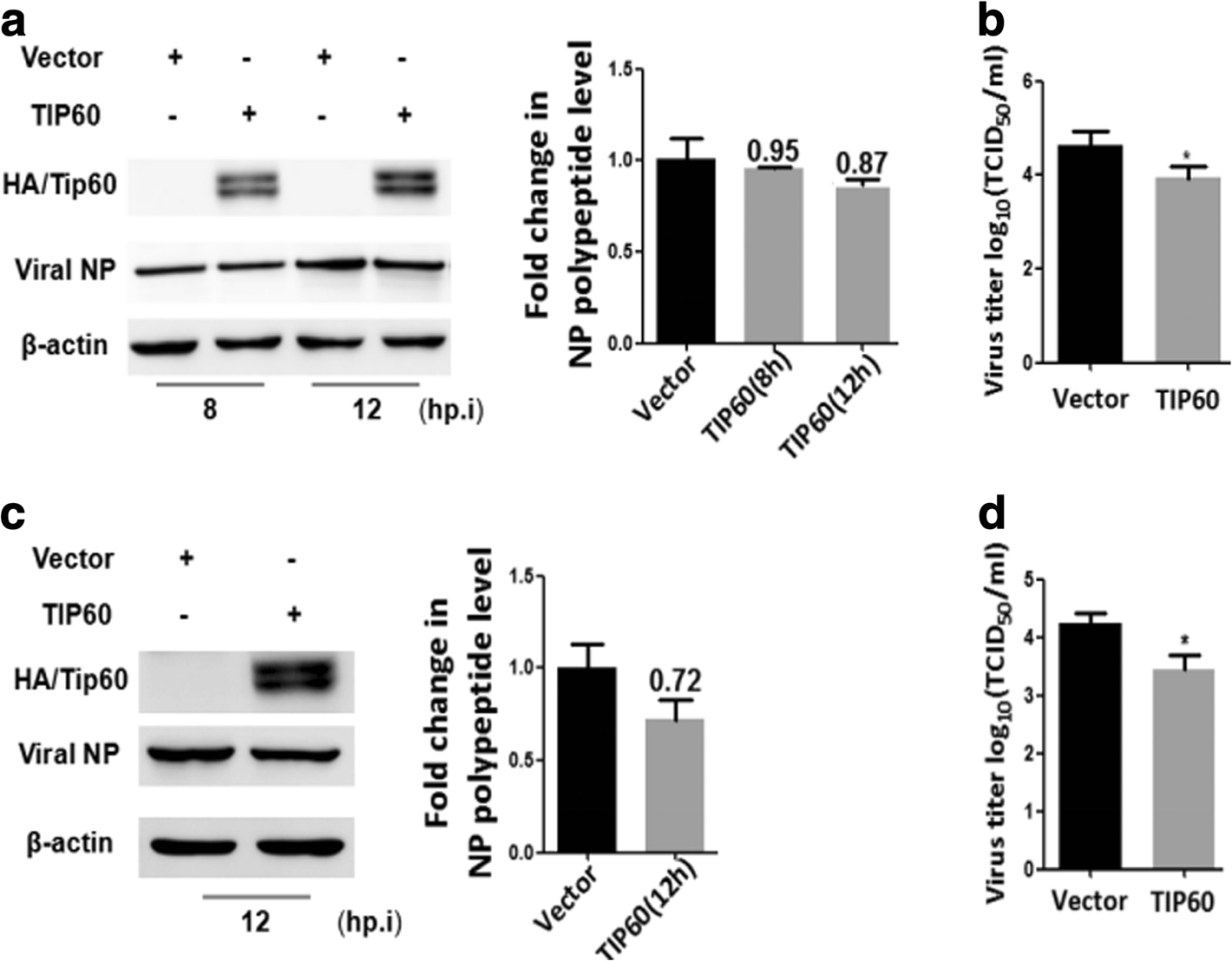
The overexpression of TIP60 inhibited IAV infection. A549 and 293 T cells (4 × 10^5^) were transfected with empty plasmid pcDNA3.0-HA (Vector) or pcDNA3.0-HA containing TIP60 (TIP60) for 48 h. Cells were then infected with IAV virus (MOI **=** 1), the culture medium and the cells were harvested separately after 8 h,12 h or only 12 h. (**a** and **c**) The cells were lysed for western blotting and detected exogenousTIP60 and NP proteins. (**b** and **d**) The infectious viral loads in the cell supernatants were determined by TCID50 analysis using 96-well plates. Data are mean **±** SD of three independent experiments. Two-way ANOVA test; *p < 0.05; **p < 0.01

To verify that TIP60 is involved in IAV infection, we infected TIP60 overexpression cells with influenza virus at an MOI of 1. The cells were lysed for western blotting and to detect exogenous TIP60 and NP protein. As shown in Fig.2a, exogenous TIP60 significantly inhibited the NP protein levels of different time points post infection. Subsequently, we examined the viral loads in the supernatants of IAV-infected cells with a TCID50 assay. The results showed that overexpression of TIP60 caused a reduction in intracellular viral yields at 12 hpi **(**Fig. 2b**)**. In addition, a similar change of NP protein levels and viral loads were obtained in IAV-infected 293 T cells at 12 hpi **(**Fig. 2c and d**)**. These results indicate that TIP60 decreased the expression of virus NP protein and reduced the production of progeny viral titer.

### TIP60 inhibited viral replication, transcription and polymerase activity

**Fig. 3:**
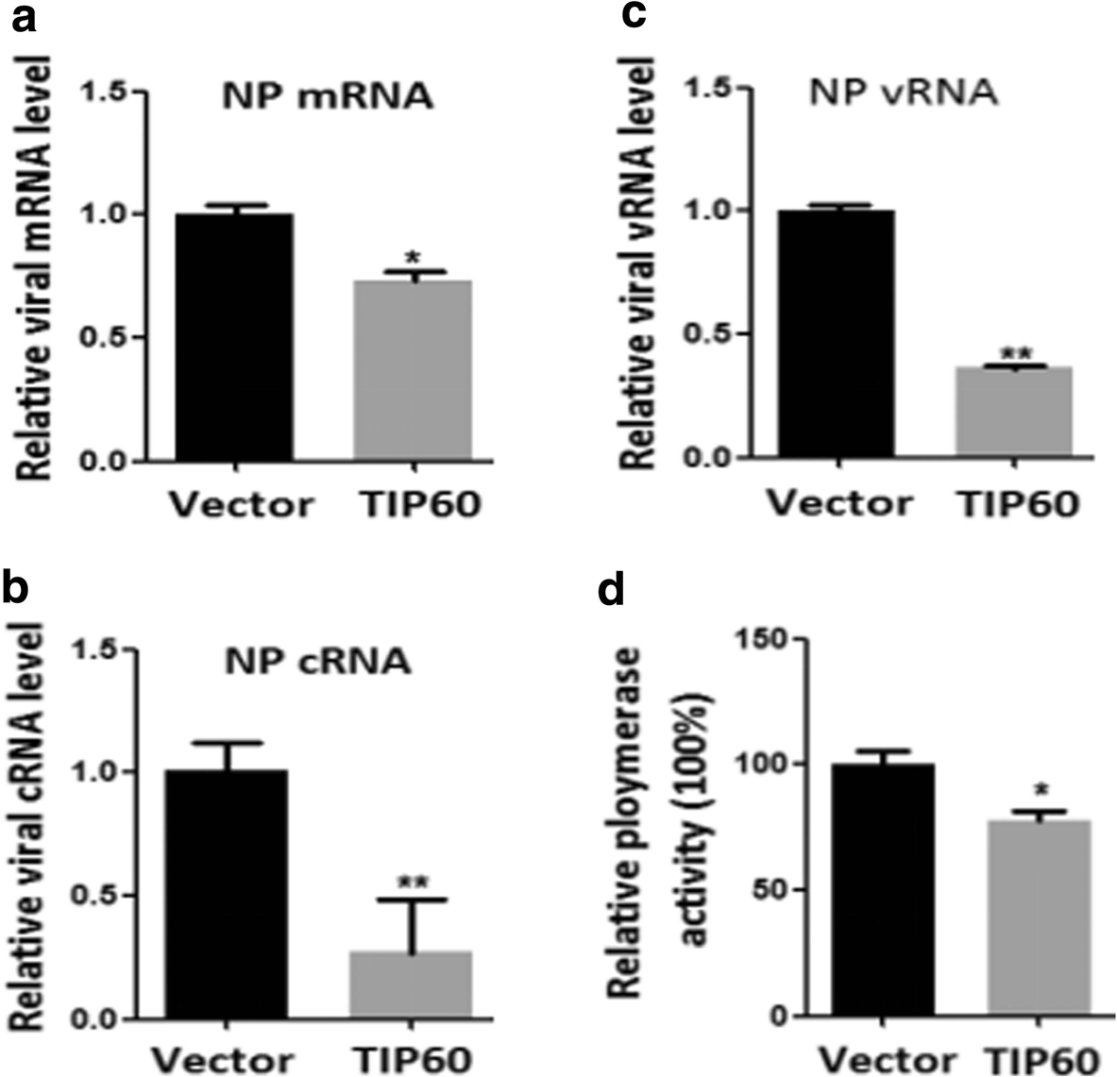
TIP60 impaired the polymerase activity and inhibited the replication and the transcription of IAV. A549 cells were transfected with empty plasmid pcDNA3.0-HA (Vector) or pcDNA3.0-HA-TIP60 (TIP60), respectively, and then infected with influenza A virus (MOI = 1). At 12 h p.i., total cellular RNA was isolated and NP mRNA (**a**), vRNA (**b**) and cRNA (**c**) levels were estimated through qRT PCR. Data are mean **±** SD of three independent experiments. Two-way ANOVA test; *p < 0.05; **p < 0.01. **d** 293 T cells were transfected with plasmids of PB2, PB1, PA and NP, together with TIP60 plasmid or control plasmid pcDNA3.0-HA. Luciferase assays were performed at 36 h after transfection**.** The data were normalized relative to the values detected in the cells transfected with pcDNA3.0-HA. Data are shown as mean ± SD (*n* = 3)

We further determined the relative expression of mRNA, vRNA and cRNA levels of NP by qRT-PCR. TIP60 overexpression resulted in a considerable decrease in NP mRNA, vRNA and cRNA levels with 0.73-, 0.36-and 0.27-fold being the maximum recorded, respectively (Figs. 3a**–**3c). Considering the inhibition of TIP60 on IAV replication, we further investigated whether TIP60 regulates IAV polymerase activity, which is essential for viral transcription and replication [9]. Luciferase assays showed that TIP60 decreases polymerase activity by 25% compared to the control **(**Fig. 3d**)**. These results indicate that TIP60 against IAV by reducing viral polymerase activity.

### TIP60 inhibited virus replication through activation TBK1-IRF3 signaling pathway

**Fig. 4:**
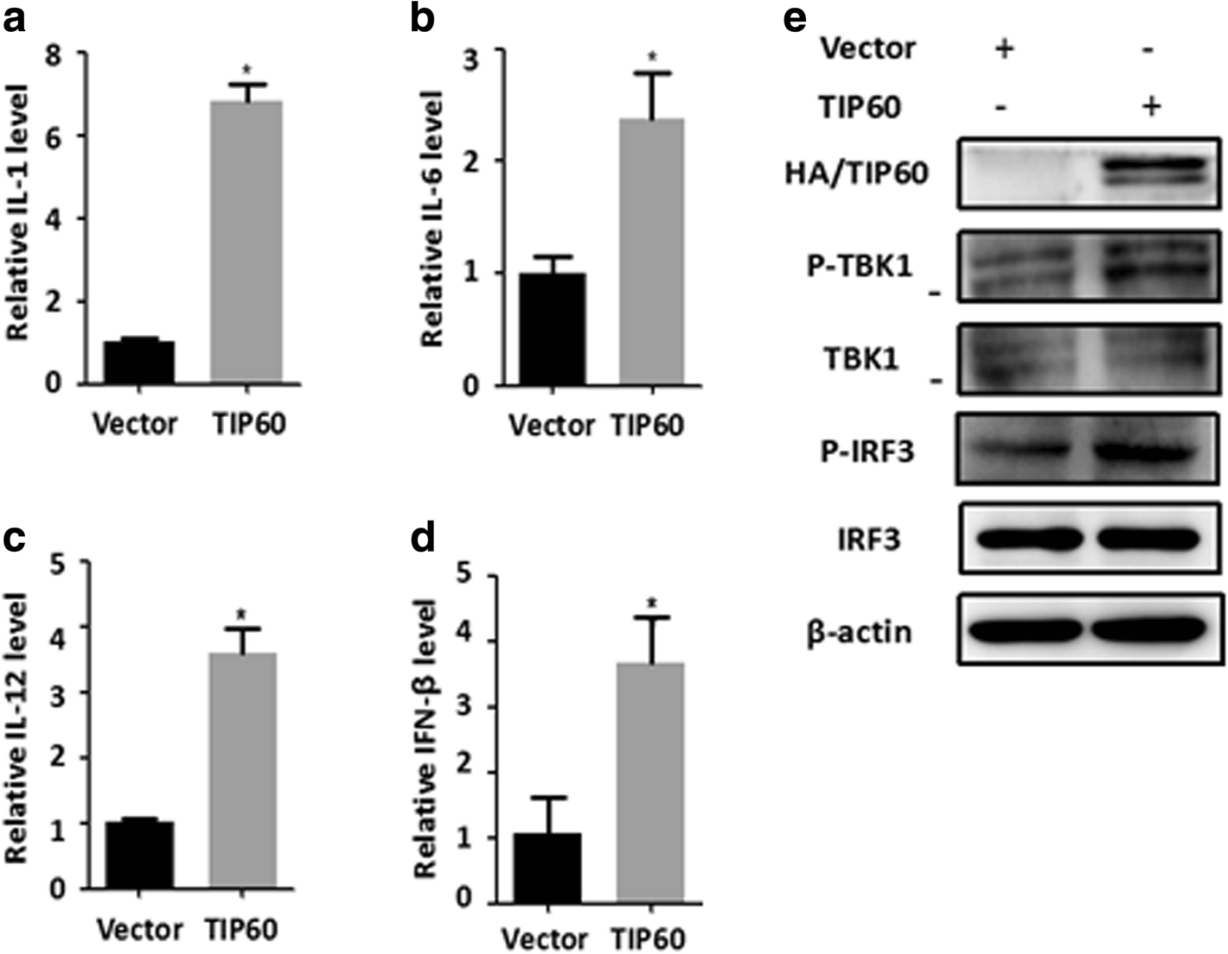
Overexpression of TIP60 inhibited Virus replication through activation TBK1-IRF3 signaling Pathway**.** A549 cells were transfected with empty plasmid pcDNA3.0-HA (Vector) or pcDNA3.0-HA containing TIP60 (TIP60) for 48 h, which were then infected with IAV virus at an MOI of 1 for 12 h. The relative expression level of IL1 (**a**), IL6 (**b**), IL12 (**c**) and IFNβ (**d**) were determined by qRT-PCR. (**e**)Total lysate of the infected cells was subjected for Western blot analysis of viral HA-TIP60, p-IRF3, IRF3, p-TBK1, TBK1. β-actin was used as a loading control. Data are mean **±** SD of three independent experiments. *p < 0.05; **p < 0.01

It has been reported that type I IFN pathway played a critical role in antivirus activity [30]. To elucidate the molecular mechanism how TIP60 regulates IAV virus production, we analyzed the expression of type I IFN and pro-inflammatory cytokines in A549 cells. A549 cells were transfected with a TIP60-expressing plasmid or an empty plasmid and subsequently infected with influenza A virus**.** Gene expression analysis using qRT-PCR showed that IL-1, IL-6 and IL-12 mRNA levels were significantly increased in TIP60-overexpression cells (Figs. 4a**–**4c). In addition, overexpression of TIP60 increased the levels of IFNβ mRNA during IAV infection (Fig. 4d). To determine whether IRF3 has a role in producing IFN type I in TIP60-overexpression cells after IAV infection, we measured the phosphorylation level of IRF3 in these cells. The results showed that phosphorylation of IRF3 were increased in TIP60-overexpression cells infected with IAVs compared with the control cells (Fig. 4e). As TBK1 has been implicated in IRF3 activation in response to viral infections, we then test whether TBK1 is involved in IRF3 activation, thus leading to type I IFN production following infection with IAV. As shown in Fig. 4e, the phosphorylation of TBK1 was increased in TIP60-overexpressioncells infected with IAV compared with the control cells. Collectively, these results suggest that overexpression of TIP60 suppresses IAV replication through activation of TBK1-IRF3 signaling pathway.

### TIP60 co-localized and interacted with NP

**Fig. 5:**
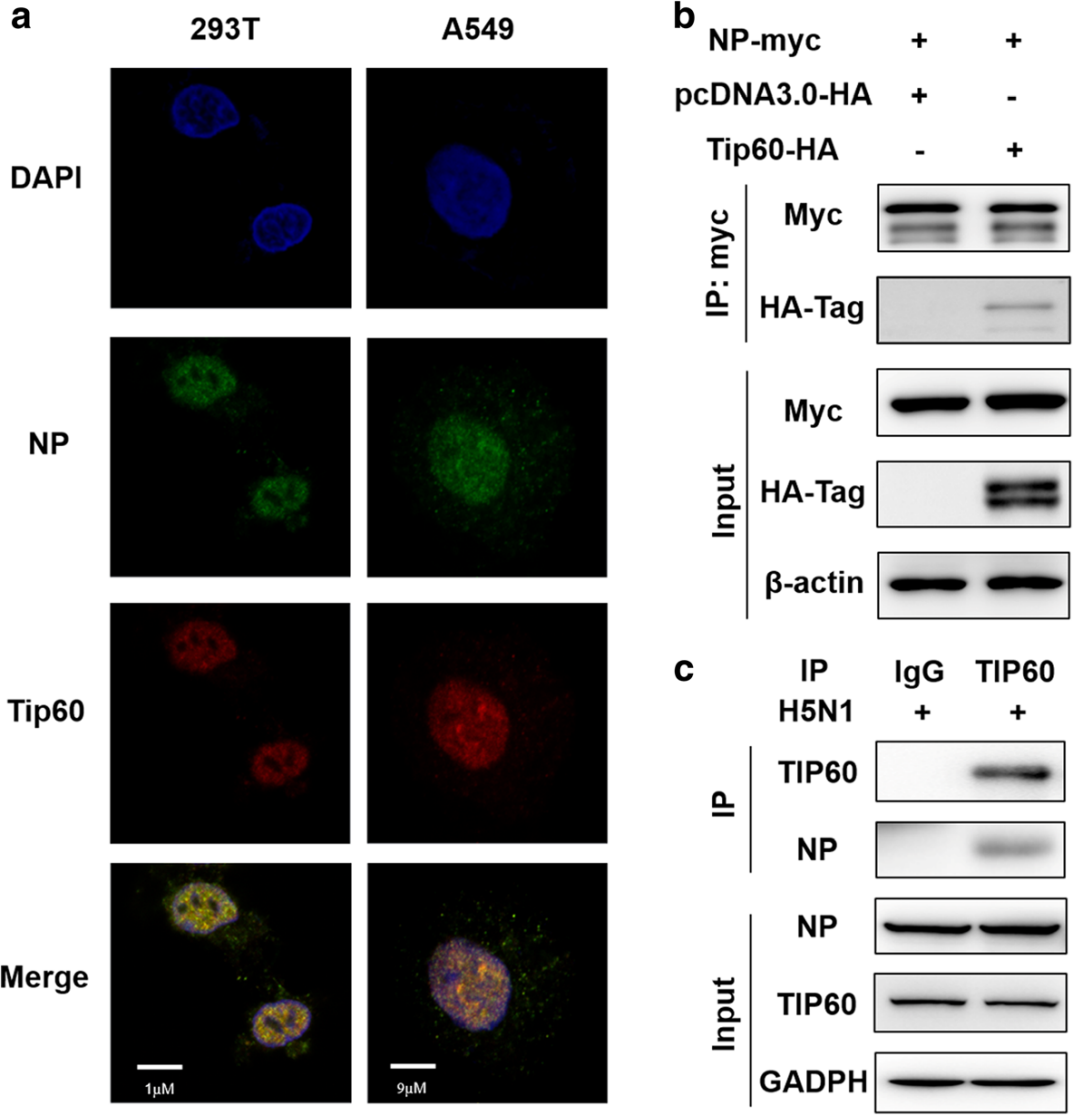
TIP60 colocalized and interacted with NP of IAV during infection. **a** A549 cells and 293 T cells were transfected with pcDNA3.0-HA-TIP60. At 48 h p.t., the cells were infected with IAV virus (MOI **=**3). At 12 h.p.i, the cells fixed and stained with DAPI (blue), anti-NP antibody (green) and anti-HA antibody (red). **b** 293 T cells were transfected with pcDNA3.1-MYC-NP and pcDNA3.0-HA-TIP60 or pcDNA3.0-HA. At 48 h post-transfection, cell lysates were immunoprecipitated with anti-MYC antibody and immunoblotted with anti-MYC or anti-HA antibodies. **c** A549 cells were infected with H5N1. After 24 h of infection, the cell lysates were subjected to immunoprecipitation with TIP60 antibody following immunoblotting using NP antibody

NP is one of the components of the viral ribonucleoprotein (vRNP) complex, and TIP60 inhibited the polymerase activity of IAV. Therefore, we investigated whether TIP60 interacts with the NP protein. We first ascertained the cellular location of TIP60 and NP by indirect immunofluorescence. A549 cells and 293 T cells were transfected with TIP60 expressing plasmids. 48 h after transfection, cells were infected with IAV at an MOI of 3 and then fixed at 12 h.p.i. Figure 5a showed that TIP60 and NP co-localize in the nucleus of A549 and 293 T cells. The TIP60**–**NP interaction was further validated by performing co-immunoprecipitation assays (Fig. 5b). These results indicate that TIP60 interacts with the NP protein and co-location in the nucleus of the host cell. To study the interaction between endogenous TIP60 and viral proteins, A549 cells were infected with H5N1. After 24 h of infection, the cell lysates were subjected to immunoprecipitation with TIP60 antibody following immunoblotting using NP antibody. The result showed that the endogenous TIP60 interacted with NP during virus infection (Fig. 5c).

## Discussion

The infection of IAV induced the expression of many genes, suggesting that some may play essential or accessory roles in the viral life cycle or the host cell’s stress response [31]. We found that IAV infection increased TIP60 mRNA as well as protein expression in A549 cells and 293 T cells. Increasing evidence suggests that TIP60 plays a role in viral life cycle. Recent research suggests that the human Papillomavirus (HPV) oncoprotein E6 interacts with and destabilizes the TIP60, and the degradation of TIP60 relieved cellular repression of the viral early promoter [26]. Also, it has been reported that the human cytomegalovirus pUL27 interacts with the TIP60 and induce its degradation by proteasome, though the effect of this degradation to the viral life cycle was unknown [27]. Another recent study showed that TIP60 binds to the early adenovirus promoter and depresses adenovirus EIA gene expression [32]. Here, we show that TIP60 overexpression resulted in a considerable decrease in NP protein and gene levels, and it reduced the progeny viral titer in cells, which is the first reported the role of TIP60 in IAV infection. We also knockdown the TIP60 in cells using siRNA and shRNA, however, the endogenous TIP60 mRNA and protein level didn’t down regulated through we repeated three times both in A549 and 293 T cells.

The replication and transcription of viral genome and mRNA in the nucleus are completed through the influenza virus polymerase complex [33]. Here, we found that the TIP60 overexpression reduced mRNA, vRNA and cRNA levels of IAV NP. In addition, we determined that TIP60 exerts its suppressive impact on the polymerase activity of IAV. Therefore, we speculated that TIP60 inhibits the replication of virus by reducing viral polymerase activity. Several proteins and compounds inhibit virus polymerase complex activity to depress the replication of influenza virus [34–37]. Moreover, we also found that TIP60 enhances the production of type I IFN and pro-inflammatory cytokines during IAV infection.

The interplay between host factors and viral proteins play important roles in host regulation of influenza virus replication. In the present study, a cellular acetyltransferase protein, TIP60, was found to interact with NP of influenza A virus. In addition, immunofluorescence analysis showed that TIP60 and IAV NP colocalized in the nucleus, suggesting that the interaction between TIP60 and NP occurs in the nucleus. NP is highly conserved among different types IAVs and it was required for the transcription and replication of influenza virus [9]. It is known to that NP protein interacts with several host factors to exhibit its diverse role in viral life cycle. Cytoskeleton-scaffolding protein actinin-4 interacts with NP to facilitate its intercompartmental localization and promotes viral replication [38]. The host protein cluster (CLU) was found to interact with NP of IAV during virus infection, and CLU overexpression attenuated NP-induced cell death and depressed IAV replication [39]. NF90 inhibits virus polymerase complex activity and replication through direct interaction with viral NP during IAV infection [36]. Interaction between MOV10 and viral Nucleoprotein inhibited the polymerase activity and virus replication [37]. The study of the interaction between host and NP can provide new ideas for the antiviral target sites [40].

## Conclusion

In conclusion, we demonstrated that IAV induced the expression of TIP60, and TIP60 inhibits influenza virus genome replication and transcription by impairs the polymerase activity. Overexpression of TIP60 suppressed replication of IAV through activated the TBK1-IRF3 pathway. In addition, we have provided evidence showing that TIP60 interacts with NP in the cell nucleus during virus infection. These findings could provide potential strategies for controlling influenza virus.

## Abbreviations

BSA: Albumin from bovine serum
cDNA: Complementary DNA
DAPI: 4′, 6-diamidino-2-phenylindole
FBS: Fetal Bovine Serum
HA: Hemagglutinin
IAV: Influenza A virus
IFN-β: Interferon-β
IL-1: Interleukin-1
IL-12: Interleukin-12
IL-6: Interleukin-6
IP: Immunoprecipitation
IRF3: Interferon regulatory factor 3
MDCK: Madin Darby canine kidney
MEM: Dulbecco’s modified eagle medium
MYST: MOZ, Ybf2/Sas3, Sas2, Tip60
NA: Neuraminidase
NP: Nucleoprotein
PA: Polymerase acidic protein
PB1: Polymerase basic protein 1
PB2: Polymerase basic protein 2
PBS: Phosphate-buffered system
PRRs: Pattern-recognition receptor
RNP: Ribonucleoprotein complexes
RT-qPCR: Reverse transcription quantitive polymerase chain reaction
SPF: Special pathogeny free
TBK1: TANK-binding kinase 1

## References

[1] Nagesh PT, Husain M (2016). Influenza a virus dysregulates host histone deacetylase 1 that inhibits viral infection in lung epithelial cells. J Virol.

[2] Zhang H, Yu H, Wang J, Zhang M, Wang X, Ahmad W, Duan M, Guan Z (2015). The BM2 protein of influenza B virus interacts with p53 and inhibits its transcriptional and apoptotic activities. Mol Cell Biochem.

[3] Chen W, Calvo PA, Malide D, Gibbs J, Schubert U, Bacik I, Basta S, O'Neill R, Schickli J, Palese P, Henklein P, Bennink JR, Yewdell JW (2001). A novel influenza a virus mitochondrial protein that induces cell death. Nat Med.

[4] Wise HM, Foeglein A, Sun J, Dalton RM, Patel S, Howard Anderson EC, Barclay WS, Digard P (2009). A complicated message: identification of a novel PB1-related protein translated from influenza a virus segment 2 mRNA. J Virol.

[5] Jagger BW, Wise HM, Kash JC, Walters KA, Wills NM, Xiao YL, Dunfee RL, Schwartzman LM, Ozinsky A, Bell GL, Dalton RM, Lo A, Efstathiou S, Atkins JF, Firth AE, Taubenberger JK, Digard P (2012). An overlapping protein-coding region in influenza a virus segment 3 modulates the host response. Science.

[6] Muramoto Y, Noda T, Kawakami E, Akkina R, Kawaoka Y (2013). Identification of novel influenza a virus proteins translated from PA mRNA. J Virol.

[7] Wise HM, Hutchinson EC, Jagger BW, Stuart AD, Kang ZH, Robb N, Schwartzman LM, Kash JC, Fodor E, Firth AE, Gog JR, Taubenberger JK, Digard P (2012). Identification of a novel splice variant form of the influenza a virus M2 ion channel with an antigenically distinct ectodomain. PLoS Pathog.

[8] Selman M, Dankar SK, Forbes NE, Jia JJ, Brown EG (2012). Adaptive mutation in influenza a virus non-structural gene is linked to host switching and induces a novel protein by alternative splicing. Emerging microbes infections.

[9] Honda A, Ueda K, Nagata K, Ishihama A (1988). RNA polymerase of influenza virus: role of NP in RNA chain elongation. The Journal of Biochemistry.

[10] Portela A, Digard P (2002). The influenza virus nucleoprotein: a multifunctional RNA-binding protein pivotal to virus replication. J Gen Virol.

[11] Batra J, Tripathi S, Kumar A, Katz JM, Cox NJ, Lal RB, Sambhara S, Lal SK (2016). Human heat shock protein 40 (Hsp40/DnaJB1) promotes influenza a virus replication by assisting nuclear import of viral ribonucleoproteins. Sci Rep.

[12] Kamine J, Elangovan B, Subramanian T, Coleman D, Chinnadurai G (1996). Identification of a cellular protein that specifically interacts with the essential cysteine region of the HIV-1 tat transactivator. Virology.

[13] Yang XJ (2004). The diverse superfamily of lysine acetyltransferases and their roles in leukemia and other diseases. Nucleic Acids Res.

[14] Sapountzi V, Logan IR, Robson CN (2006). Cellular functions of TIP60. The international journal of biochemistry cell biology.

[15] McAllister D, Merlo X, Lough J (2002). Characterization and expression of the mouse tat interactive protein 60 kD (TIP60) gene. Gene.

[16] Patel JH, Du Y, Ard PG, Phillips C, Carella B, Chen CJ, Rakowski C, Chatterjee C, Lieberman PM, Lane WS, Blobel GA, McMahon SB (2004). The c-MYC oncoprotein is a substrate of the acetyltransferases hGCN5/PCAF and TIP60. Mol Cell Biol.

[17] Xiao H, Chung J, Kao HY, Yang YC (2003). TIP60 is a co-repressor for STAT3. J Biol Chem.

[18] Sun Y, Jiang X, Chen S, Fernandes N, Price BD (2005). A role for the TIP60 histone acetyltransferase in the acetylation and activation of ATM. Proc Natl Acad Sci U S A.

[19] Tang Y, Luo J, Zhang W, Gu W (2006). TIP60-dependent acetylation of p53 modulates the decision between cell-cycle arrest and apoptosis. Mol Cell.

[20] Legube G, Linares LK, Tyteca S, Caron C, Scheffner M, Chevillard-Briet M, Trouche D (2004). Role of the histone acetyl transferase TIP60 in the p53 pathway. J Biol Chem.

[21] Xu L, Chen Y, Song Q, Xu D, Wang Y, Ma D (2009). PDCD5 interacts with TIP60 and functions as a cooperator in acetyltransferase activity and DNA damage-induced apoptosis. Neoplasia.

[22] Liu N, Wang J, Wang J, Wang R, Liu Z, Yu Y, Lu H (2013). ING5 is a TIP60 cofactor that acetylates p53 in response to DNA damage. Cancer Res.

[23] Dar A, Shibata E, Dutta A (2013). Deubiquitination of TIP60 by USP7 determines the activity of the p53-dependent apoptotic pathway. Mol Cell Biol.

[24] Takino T, Nakada M, Li Z, Yoshimoto T, Domoto T, Sato H (2016). TIP60 regulates MT1-MMP transcription and invasion of glioblastoma cells through NF-κB pathway. Clinical & experimental metastasis.

[25] Awasthi S, Sharma A, Wong K, Zhang J, Matlock EF, Rogers L, Motloch P, Takemoto S, Taguchi H, Cole MD, Luscher B, Dittrich O, Tagami H, Nakatani Y, McGee M, Girard AM, Gaughan L, Robson CN, Monnat RJ, Harrod J (2005). A human T-cell lymphotropic virus type 1 enhancer of Myc transforming potential stabilizes Myc-TIP60 transcriptional interactions. Mol Cell Biol.

[26] Jha S, Pol SV, Banerjee NS, Dutta AB, Chow LT, Dutta A (2010). Destabilization of TIP60 by human papillomavirus E6 results in attenuation of TIP60-dependent transcriptional regulation and apoptotic pathway. Mol Cell.

[27] Reitsma JM, Savaryn JP, Faust K, Sato H, Halligan BD, Terhune SS (2011). Antiviral inhibition targeting the HCMV kinase pUL97 requires pUL27-dependent degradation of TIP60 acetyltransferase and cell-cycle arrest. Cell Host Microbe.

[28] Chen L, Wang C, Luo J, Li M, Liu H, Zhao N, Huang J, Zhu X, Ma G, Yuan G, He H (2017). Amino acid substitution K470R in the nucleoprotein increases the virulence of H5N1 influenza a virus in mammals. Front Microbiol.

[29] Livak KJ, Schmittgen TD (2001). Analysis of relative gene expression data using real-time quantitative PCR and the 2− ΔΔCT method. methods.

[30] Ehrhardt C, Seyer R, Hrincius ER, Eierhoff T, Wolff T, Ludwig S (2010). Interplay between influenza a virus and the innate immune signaling. Microbes Infect.

[31] Geiss GK, An MC, Bumgarner RE, Hammersmark E, Cunningham D, Katze MG (2001). Global impact of influenza virus on cellular pathways is mediated by both replication-dependent and-independent events. J Virol.

[32] Gupta A, Jha S, Engel DA, Ornelles DA, Dutta A (2013). TIP60 degradation by adenovirus relieves transcriptional repression of viral transcriptional activator EIA. Oncogene.

[33] Perez JT, Zlatev I, Aggarwal S, Subramanian S, Sachidanandam R, Kim B, Manoharan M (2012). A small-RNA enhancer of viral polymerase activity. J Virol.

[34] Yu J, Wang D, Jin J, Xu J, Li M, Wang H, Dou J, Zhou C (2016). Antiviral activity of SA-2 against influenza a virus in vitro/vivo and its inhibition of RNA polymerase. Antivir Res.

[35] Sasaki Y, Kakisaka M, Chutiwitoonchai N, Tajima S, Hikono H, Saito T, Aida Y (2014). Identification of a novel multiple kinase inhibitor with potent antiviral activity against influenza virus by reducing viral polymerase activity. Biochem Biophys Res Commun.

[36] Wang P, Song W, Mok BW, Zhao P, Qin K, Lai A, Smith GJ, Zhang J, Lin T, Guan Y, Chen H (2009). Nuclear factor 90 negatively regulates influenza virus replication by interacting with viral nucleoprotein. J Virol.

[37] Zhang J, Huang F, Tan L, Bai C, Chen B, Liu J, Liang J, Liu C, Zhang S, Lu G, Chen Y, Zhang H (2016). Host protein Moloney leukemia virus 10 (MOV10) acts as a restriction factor of influenza a virus by inhibiting the nuclear import of the viral nucleoprotein. J Virol.

[38] Sharma S, Mayank AK, Nailwal H, Tripathi S, Patel JR, Bowzard JB, Gaur P, Donis RO, Katz JM, Cox NJ, Lal RB, Farooqi H, Sambhara S, Lal SK (2014). Influenza a viral nucleoprotein interacts with cytoskeleton scaffolding protein α-actinin-4 for viral replication. FEBS J.

[39] Tripathi S, Batra J, Cao W, Sharma K, Patel JR, Ranjan P, Kumar A, Katz JM, Cox NJ, Lal RB, Sambhara S (2013). Influenza a virus nucleoprotein induces apoptosis in human airway epithelial cells: implications of a novel interaction between nucleoprotein and host protein Clusterin. Cell death disease.

[40] Kao RY, Yang D, Lau LS, Tsui WH, Hu L, Dai J, Chan MP, Chan CM, Wang P, Zheng BJ, Sun J (2010). Identification of influenza a nucleoprotein as an antiviral target. Nat Biotechnol.

